# Remnant Cholesterol as a Predictor of Target-Vessel Failure in Patients With In-Stent Restenosis

**DOI:** 10.31083/RCM43867

**Published:** 2025-11-26

**Authors:** Xiao-han Kong, Zi-han Lv, Yi-fei Wang, Yin-dong Sun, Tian Xu, Wei You, Pei-na Meng, Xiang-qi Wu, Zhi-ming Wu, Hai-bo Jia, Fei Ye

**Affiliations:** ^1^Department of Geriatrics, Nanjing First Hospital, Nanjing Medical University, 210006 Nanjing, Jiangsu, China; ^2^Department of Cardiology, Nanjing First Hospital, Nanjing Medical University, 210006 Nanjing, Jiangsu, China

**Keywords:** remnant cholesterol, percutaneous coronary intervention, in-stent restenosis

## Abstract

**Background:**

The clinical value of remnant cholesterol (RC) in patients with in-stent restenosis (ISR) who undergo percutaneous coronary intervention (PCI) is unknown. Therefore, this study aimed to clarify the association between increased RC levels and clinical prognosis in patients with ISR.

**Methods:**

This retrospective study enrolled 836 patients diagnosed with ISR. The study population was divided into four quartiles (Q1–Q4) according to median RC levels. Using a multivariate Cox proportional hazards model and Kaplan–Meier (KM) curve, the association between RC levels and the study endpoint, defined as target-vessel failure (TVF) within 3 years after PCI, was investigated. A discordance analysis was also performed with several definitions.

**Results:**

The KM curve showed an increased risk of TVF with elevated RC levels (*p* < 0.001). After adjustment, the RC level was identified as an independent predictor of TVF, regardless of whether the metric was considered as a continuous or categorical variable (hazard ratio (HR) = 1.37, 95% confidence interval (CI): 1.16–1.62; *p* < 0.001; HR = 3.43, 95% CI: 1.85–6.36; *p* < 0.001). Subgroup analysis showed that the RC-related TVF risk was more pronounced in patients with low-density lipoprotein cholesterol (LDL-C) <1.8 mmol/L (2.75 for each one standard deviation (SD) increase, 95% CI: 1.66–4.55; *p* for interaction < 0.001). In the discordance analysis, individuals with discordantly high RC levels rather than high LDL-C levels had an increased risk of TVF (HR = 2.02, 95% CI: 1.33–3.07; *p* < 0.001).

**Conclusions:**

An increased RC level was associated with an elevated risk of TVF in patients with ISR who underwent PCI. Further, the RC-related risk was more pronounced in patients with LDL-C levels <1.8 mmol/L.

## 1. Introduction

Arteriosclerotic cardiovascular disease (ASCVD) remains the primary cause of cardiac mortality worldwide [1]. Early revascularization and pharmacotherapy have improved clinical prognosis among patients with coronary artery disease [2]. As a stent failure event, in-stent restenosis (ISR) still occurs at an annual incidence rate of 1–2% even with the utilization of the new generation of drug-eluting stents (DES) [3, 4]. Compared with de novo lesions, patients with ISR have an increased cardiovascular risk, such as a higher incidence of post-percutaneous coronary intervention (PCI) myocardial infarction (MI) and repetitive target vessel revascularization (TVR) [5, 6]. Thus, risk classification and management in this population are important.

As one of the cornerstone treatments for ASCVD, Drugs that aim to reduce low-density lipoprotein cholesterol (LDL-C) have demonstrated considerable effects in stabilizing plaques and promoting plaque regression [7]. However, there are still several patients who experience residual risks for cardiac events even with adequate LDL-C control [8, 9]. Owing to this, researchers have recently shifted to non-LDL-C control because of the persistent residual cardiac risk, even in individuals who have undergone various lipid-lowering therapies [10, 11, 12, 13, 14].

Remnant cholesterol (RC), the cholesterol content within triglyceride-rich lipoproteins, has been established as a risk factor for ASCVD in primary or secondary prevention [15, 16]. In patients with ISR, the clinical value of RC levels on cardiovascular outcomes has not been clarified. Therefore, this study aimed to assess the association between increased RC levels and cardiac events among patients with ISR who underwent re-PCI and to evaluate its independent influence among different LDL-C levels.

## 2. Methods

### 2.1 Study Population

**Fig. 1.**
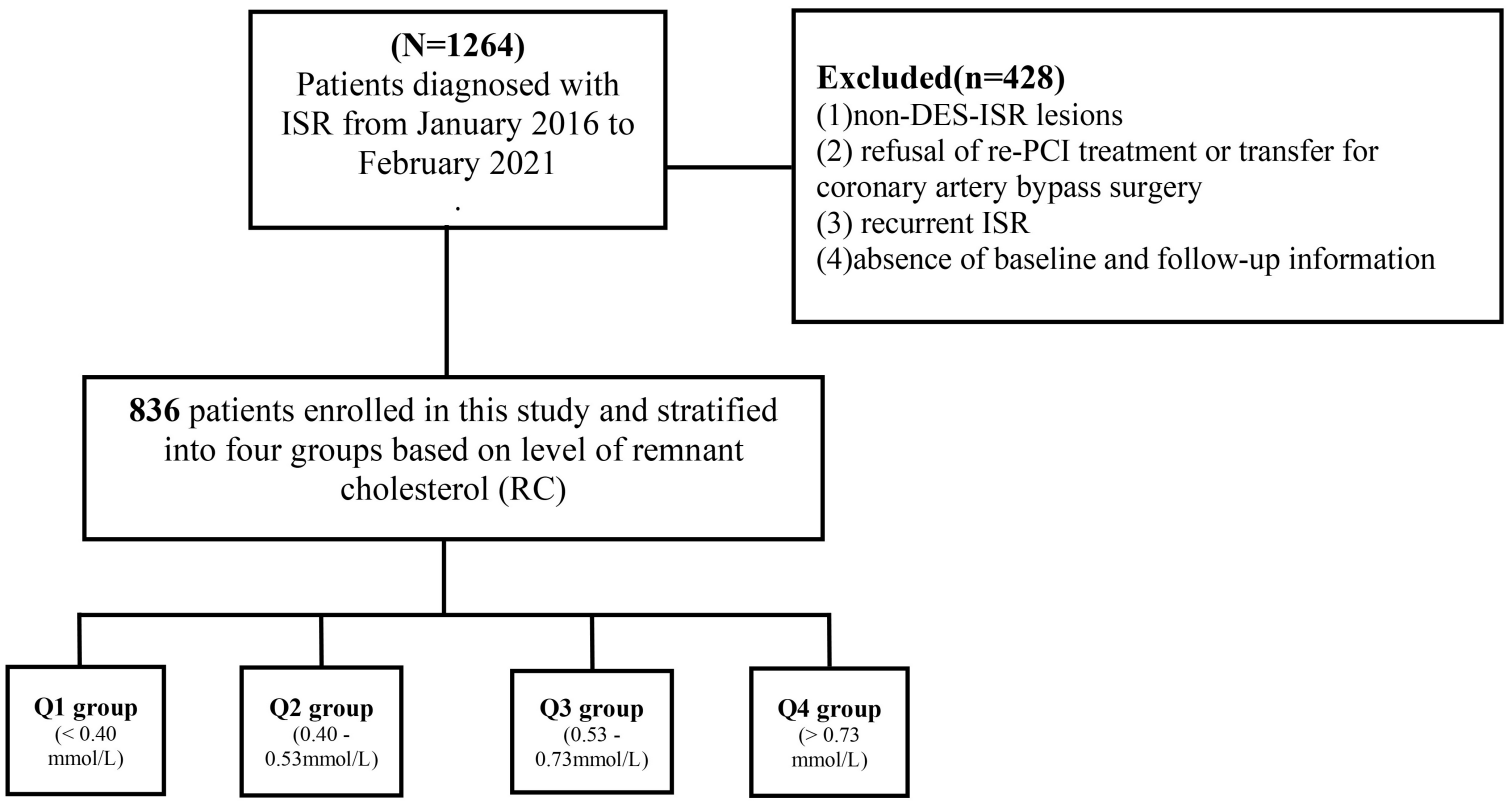
**Study flowchart**. Abbreviations: DCB, drug-coated balloon; DES, drug-eluting stent; ISR, in-stent restenosis; RC, remnant cholesterol; PCI, percutaneous coronary intervention.

This retrospective study included patients diagnosed with DES-ISR at Nanjing First Hospital from January 2016 to February 2021. ISR was defined as a reduction of ≥50% in the vessel diameter in the previously stented segment, as assessed by coronary angiography [17, 18]. The exclusion criteria were as listed: (1) non-DES-ISR lesions, (2) refusal of re-PCI treatment or transfer for coronary artery bypass surgery, (3) recurrent ISR, and (4) absence of baseline or follow-up information (Fig. 1).

### 2.2 Data Collection

Demographic and clinical characteristics were retrieved from the electronic medical system at Nanjing First Hospital. Fasting venous blood samples were procured at fixed time intervals and then analyzed at the central laboratory. The level of RC was calculated as follows: RC level (mmol/L) = total cholesterol (TC) level (mmol/L) minus high-density lipoprotein cholesterol (HDL-C) level (mmol/L) minus LDL-C level (mmol/L) [19]. Body mass index (BMI) was defined as weight (kg)/height (m^2^). Patients with a history of type 2 diabetes mellitus, receiving hypoglycemic treatment, or individuals presenting with typical symptoms of diabetes, such as fasting blood glucose >7 mmol/L or hemoglobin A1C ≥6.5%, were categorized as having diabetes [20]. Hypertension was defined as an average blood pressure ≥140/90 mmHg based on three repetitive measurements or a self-reported prior diagnosis [21].

### 2.3 Discordance Definition

To further investigate the RC-associated risk beyond LDL-C level, this study conducted discordance analyses. First, discordance was defined as a difference greater than 10 percentile units (RC percentile minus LDL-C percentile). The study population was then divided into: (1) RC percentile < LDL-C percentile by >10 percentile units (discordantly low RC/high LDL-C); (2) concordant RC/LDL-C within ± 10 percentile units; and (3) RC percentile > LDL-C percentile by >10 percentile units (discordantly high RC/low LDL-C). Second, we used the absolute value of the clinical cutoff points to define the discordance relationship between RC and LDL-C. According to previous guidelines and recommendations, this study focused on different LDL-C cutoff points (2.6 mmol/L and 1.8 mmol/L), and the RC cutoff point was defined as 0.8 mmol/L [22, 23].

### 2.4 Study Outcomes

The primary outcome of this research was target-vessel failure (TVF) during the follow-up period after PCI, which was defined as a composite of MI, TVR, and all-cause mortality [24]. The secondary outcome included each component of the primary outcome. MI was identified based on the third Universal Definition of Myocardial Infarction [25]. TVR was characterized as revascularization that was either angina/ischemia-induced or clinically motivated, including PCI or coronary artery bypass grafting [26, 27]. Clinical follow-up was conducted at 3–6 months intervals throughout the 3-year follow-up period. This work was carried out by independent clinical research coordinators who remained blinded to the study objective and research data.

### 2.5 Statistical Analysis

Continuous variables that follow a normal distribution are presented in the form of means ± standard deviations. For non-normally distributed variables, the median and interquartile range (IQR) are employed for description. Categorical variables are expressed as percentages (%). The *t*-, Kruskal–Wallis, and chi-squared tests were used for data analysis. The study population were divided into four quartiles (Q1–Q4) based on RC levels [(Q1), <0.40; (Q2), 0.40–0.53; (Q3), 0.54–0.73; (Q4), >0.73)]. To calculate the association between RC level and study endpoint, we constructed a Cox proportional hazard model. Baseline variables deemed clinically relevant or demonstrating a univariate association (*p* value < 0.05) with the endpoint were included in the multivariate model. Finally, the multivariate analysis was adjusted for sex, age, diabetes, hypertension, N-terminal pro-B-type natriuretic peptide (NT-pro BNP), lipoprotein A, red blood cell, triglycerides, and serum creatinine. These results are reported as hazard ratio (HR) along with 95% confidence interval (CI). Subsequently, the stability of the association between RC level and follow-up TVF was examined by subgroup analysis. RC values were Z-score-standardized before regression analysis because of a non-normal distribution. When counted as a continuous variable, the impact of RC on the primary endpoint was evaluated using the per-standard deviation (SD) increase format. A Kaplan–Meier (KM) curve was used to show the occurrence of TVF among different quartiles using the log-rank test. Further, this study performed discordance analyses between RC and LDL-C using several approaches. Lastly, we used receiver operating characteristic (ROC) analysis to estimate the diagnostic value of RC level, LDL-C level, and RC/LDL-C discordance. The area under the ROC curve (AUC) of the different models was compared to evaluate the incremental effect of RC on risk assessment. The values of integrated discrimination improvement (IDI) and net reclassification improvement (NRI) were computed to estimate the incremental predictive value. All statistical analyses were conducted using R software (version 4.2.1, R Core Team, Vienna, Austria). Statistical significance was defined as a two-sided *p*-value < 0.05.

## 3. Results

### 3.1 Baseline Characteristics

This study enrolled 836 individuals diagnosed with ISR. The average age was 66.22 ± 10.51, and 662 (79.2%) were male. Individuals with elevated RC levels had a higher BMI, a greater incidence of diabetes and MI, and were mostly female. Additionally, patients in higher RC quartiles had greater levels of TC, LDL-C, triglycerides, and fasting blood glucose. Individuals across different quartiles had comparable lesion and procedural features, including target lesion location, average stent diameter, and total stent length (*p* all > 0.05). Almost all patients underwent statin therapy after PCI (98.2%); in some cases, it was combined with ezetimibe (38.2%) or a PCSK9 inhibitor (4.6%) (Table 1).

**Table 1.** **Baseline characteristics in patients with different quantiles of RC**.

Variables			Total (n = 836)	Q1 (n = 209)	Q2 (n = 209)	Q3 (n = 209)	Q4 (n = 209)	*p*
Age			66.22 ± 10.51	65.98 ± 10.79	66.61 ± 10.69	67.16 ± 10.56	65.14 ± 9.96	0.236
BMI			25.09 ± 3.30	24.79 ± 3.19	24.71 ± 3.45	25.18 ± 2.94	25.70 ± 3.50	0.007
Male, n (%)			662 (79.2)	185 (88.5)	164 (78.5)	161 (77.0)	152 (72.7)	<0.001
Disease history								
	Hypertension, n (%)		623 (74.5)	156 (74.6)	154 (73.7)	155 (74.2)	158 (75.6)	0.974
	Diabetes, n (%)		318 (38.0)	69 (33.0)	80 (38.3)	63 (30.1)	106 (50.7)	0.001
	Smoking, n (%)		421 (50.4)	105 (50.2)	102 (48.8)	106 (50.7)	108 (51.7)	0.996
Diagnosis								
	Stable angina		138 (16.5)	33 (15.8)	38 (18.2)	35 (16.8)	32 (15.3)	0.866
	Unstable angina		532 (63.6)	150 (71.8)	129 (61.7)	138 (66.0)	115 (55.0)	0.004
	Myocardial infarction		166 (19.9)	26 (12.4)	42 (20.1)	36 (17.2)	62 (29.7)	<0.001
Lesion and procedure characteristics								
	Target lesion location, n (%)							0.054
		LAD	497 (59.5)	116 (55.5)	144 (68.9)	120 (57.4)	117 (56.0)	
		LCX	88 (10.5)	27 (12.9)	19 (9.1)	19 (9.1)	23 (11.0)	
		RCA	251 (30.0)	66 (31.6)	46 (22.0)	70 (33.5)	69 (33.0)	
	Average stent diameters, mm		2.93 ± 0.61	2.89 ± 0.69	2.96 ± 0.51	2.90 ± 0.69	2.98 ± 0.55	0.325
	Total length of stents, mm		31.00 (18.00, 54.00)	29.00 (18.00, 47.00)	30.00 (18.00, 52.00)	30.00 (18.00, 52.00)	36.00 (18.00, 56.00)	0.067
Laboratory tests								
	RC, mmol/L		0.54 (0.40, 0.73)	0.31 (0.25, 0.35)	0.47 (0.44, 0.50)	0.62 (0.58, 0.67)	0.94 (0.81, 1.16)	<0.001
	TC, mmol/L		3.36 (2.85, 4.08)	2.84 (2.56, 3.37)	3.13 (2.76, 3.56)	3.49 (3.04, 4.10)	4.30 (3.66, 5.15)	<0.001
	LDL-C, mmol/L		1.78 (1.40, 2.39)	1.59 (1.26, 2.01)	1.69 (1.32, 2.06)	1.83 (1.44, 2.42)	2.32 (1.76, 3.04)	<0.001
	HDL-C, mmol/L		0.94 (0.81, 1.10)	0.96 (0.84, 1.16)	0.95 (0.83, 1.12)	0.96 (0.81, 1.10)	0.91 (0.76, 1.03)	0.007
	Triglycerides, mmol/L		1.34 (0.96, 1.85)	0.92 (0.74, 1.16)	1.20 (0.93, 1.48)	1.40 (1.10, 1.69)	2.27 (1.77, 3.09)	<0.001
	WBC, 10^9^/L		6.56 (5.44, 7.83)	6.30 (5.37, 7.35)	6.69 (5.62, 8.14)	6.52 (5.37, 7.79)	6.68 (5.47, 8.07)	0.083
	RBC, 10^12^/L		4.40 (4.04, 4.77)	4.45 (4.11, 4.76)	4.37 (4.06, 4.72)	4.45 (4.04, 4.80)	4.38 (3.97, 4.80)	0.595
	FBG, mmol/L		5.61 (4.94, 7.14)	5.39 (4.80, 6.81)	5.43 (4.89, 6.94)	5.43 (4.89, 6.59)	6.29 (5.29, 8.84)	<0.001
	NT-pro BNP		311.40 (100.00, 969.00)	246.14 (100.00, 536.03)	351.60 (129.94, 877.47)	429.85 (145.98, 1971.01)	279.77 (100.00, 969.00)	0.001
	Scr		75.45 (63.38, 92.00)	75.00 (64.00, 87.00)	72.00 (62.00, 87.00)	79.00 (65.10, 96.00)	77.00 (64.00, 96.00)	0.008
Meditation								
	Statin, n (%)		821 (98.2)	205 (98.1)	208 (99.5)	207 (99.0)	201 (96.2)	0.081
	Ezetimibe, n (%)		319 (38.2)	66 (31.6)	88 (42.1)	84 (40.2)	81 (38.8)	0.132
	PCSK9-inhibitor, n (%)		38 (4.6)	12 (5.7)	8 (3.8)	6 (2.9)	12 (5.7)	0.395

BMI, body mass index; FBG, fasting Blood Glucose; HDL-C, high-density lipoprotein cholesterol; LAD, left anterior descending; LCX, left circumflex; LDL-C, low-density lipoprotein cholesterol; PCSK9, proprotein convertase subtilisin kexin/type 9; RBC, red blood cell; RC, remnant cholesterol; RCA, right coronary artery; Scr, Serum creatinine; TC, Total cholesterol; WBC, white blood cell; NT-pro BNP, N-terminal pro-B-type natriuretic peptide.

### 3.2 Associations Between RC Level and TVF

**Fig. 2.**
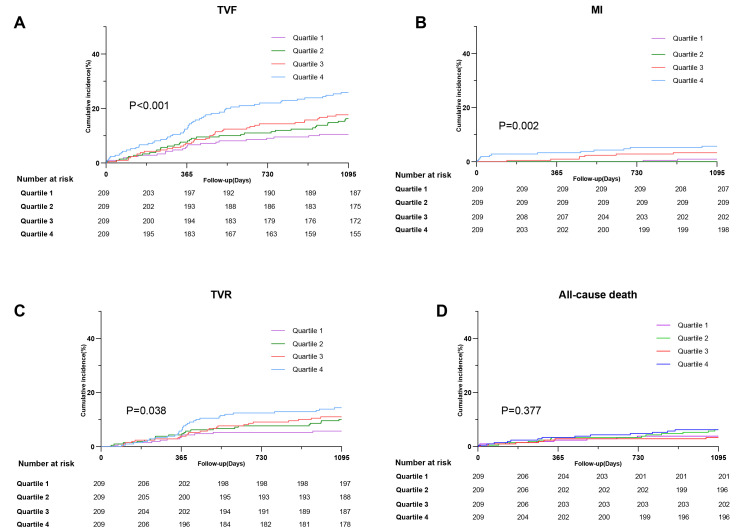
**Incidence of clinical outcomes in patients with ISR with different quantiles of RC (A) TVF (B) MI (C) TVR (D) All-cause death**. Abbreviations: ISR, in-stent restenosis; MI, myocardial infarction; TVR, target vessel revascularization; TVF, target-vessel failure.

Over the 3 years of follow-up, 147 (17.6%) patients experienced TVF. The KM curve showed significant differences among the four quartiles (Q1–Q4), with individuals in the highest quartile having an elevated incidence of TVF compared with others (10.5% vs. 16.3% vs. 17.7% vs. 25.8%; *p* < 0.001) (Fig. 2A). For each component of adverse events, the incidence of MI and TVR rise significantly in the higher RC quartiles (*p* = 0.002; *p* = 0.038) (Fig. 2B,C). However, patients in the highest RC quartile did not show an increase in the rate of all-cause mortality compared to those in the other quartiles (*p* = 0.377) (Fig. 2D).

A Cox regression model was used to explore the relation between RC level and TVF (Table 2). In the univariate analysis, RC level was significantly associated with the risk of TVF, whether considered as a continuous or categorical variable. After adjusting for confounding factors, multivariable Cox analysis revealed that RC level was associated with TVF risk (1.37 for each 1 SD increase, 95% CI: 1.16–1.62; *p* < 0.001). When considered a categorical variable, the Q4 quartile showed a 3.43-fold (95% CI: 1.85–6.36) higher risk of TVF than the Q1 quartile.

**Table 2.** **Cox regression model for the association between the RC and TVF**.

Variables		Univariate Cox regression model		Multivariable Cox regression model	
		*p*	HR (95% CI)	*p*	HR (95% CI)
Continuous					
	RC level (mmol/L), per 1 SD increase	<0.001	1.19 (1.09∼1.29)	<0.001	1.37 (1.16∼1.62)
Categorical					
	Q1 quartile	(Reference)	1.00	(Reference)	1.00
	Q2 quartile	0.096	1.58 (0.92∼2.70)	0.047	1.99 (1.01∼3.94)
	Q3 quartile	0.042	1.73 (1.02∼2.93)	0.144	1.65 (0.84∼3.25)
	Q4 quartile	<0.001	2.74 (1.67∼4.49)	<0.001	3.43 (1.85∼6.36)

Adjusted for sex, age, diabetes, hypertension, NT-pro BNP, Lp(a), RBC, triglycerides, and Scr. RBC, red blood cell; RC, remnant cholesterol; Scr, Serum creatinine; TVF, target-vessel failure; Lp(a), Lipoprotein (a); HR, hazard ratio; CI, confidence interval.

### 3.3 Subgroup Analysis

**Fig. 3.**
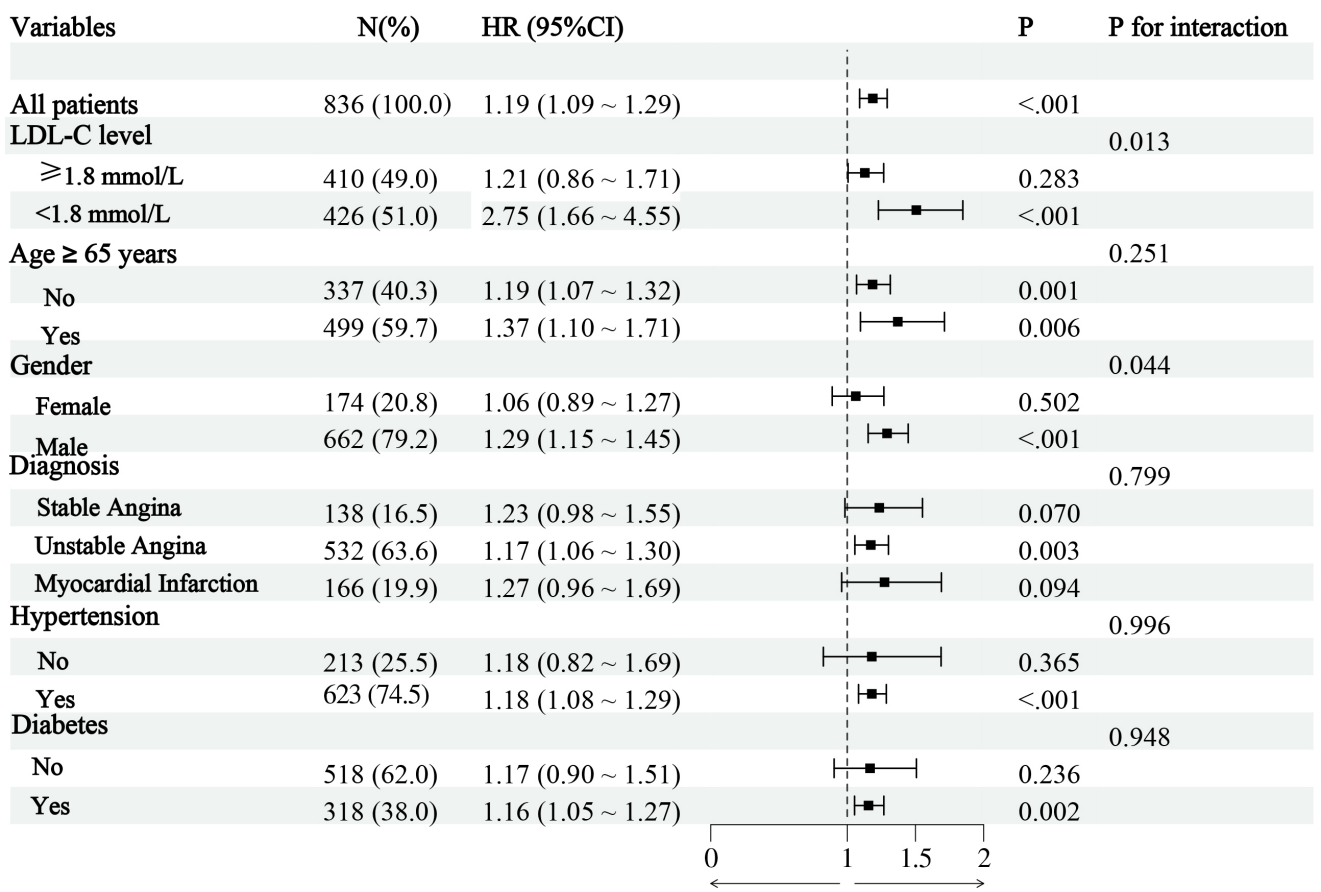
**Subgroup analysis**. Abbreviations: LDL-C, low-density lipoprotein cholesterol.

To further clarify the influence of RC on post-PCI TVF, we conducted a subgroup analysis (Fig. 3). The results showed no statistically significant interactions between age, diagnosis, hypertension, and diabetes (all *p* for interaction > 0.05). Compared with females, males with elevated RC levels demonstrated a marginally increased risk of TVF (1.29 for each 1 SD increase, 95% CI: 1.15–1.45; *p* < 0.001) (*p* for interaction = 0.044). Further, a significant difference in RC-related risk was observed in the subgroup of LDL-C levels (1.8 mmol/L). For patients with lower LDL-C level (<1.8 mmol/L), the HR (95% CI) of TVF was 2.75 (1.66–4.55), compared with no significant residual risk in patients with LDL-C ≥1.8 mmol/L (1.21 for each 1 SD increase, 95% CI: 0.86–1.71; *p* = 0.283) (*p* for interaction = 0.013) (Fig. 3).

### 3.4 Discordance Analyses of RC and LDL-C Levels

**Fig. 4.**
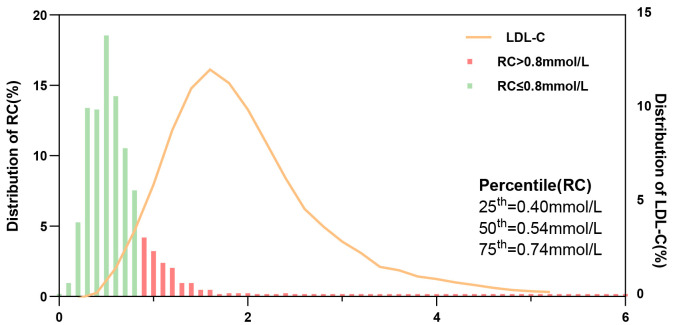
**Distribution of RC and LDL-C levels in patients with ISR**. Abbreviations: ISR, in-stent restenosis; RC, remnant cholesterol; LDL-C, low-density lipoprotein cholesterol.

Fig. 4 shows the distribution of RC and LDL-C levels. The median remnant-C level was 0.54 mmol/L (IQR, 0.40–0.73 mmol/L), and 160 (19.1%) were identified as having a high RC level (>0.8 mmol/L). Further, for LDL-C level, almost half of the individuals (49.0%) had a LDL-C level ≥1.8 mmol/L, and 147 (17.6%) had a level ≥2.6 mmol/L.

**Fig. 5.**
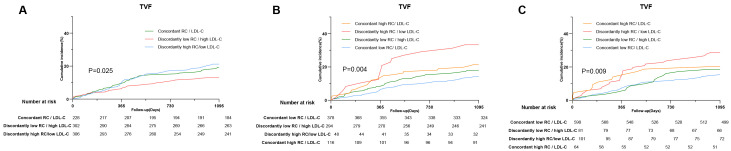
**Discordance analyses of RC/LDL-C levels for the incidence of TVF**. (A) Discordance is defined by percentiles. (B) Discordance is defined by clinical cutoff value (RC: 0.8 mmol/L; LDL-C: 1.8 mmol/L). (C) Discordance is defined by clinical cutoff value (RC: 0.8 mmol/L; LDL-C: 2.6 mmol/L). Abbreviations: RC, remnant cholesterol; LDL-C, low-density lipoprotein cholesterol.

In the discordance analysis, when identified by percentiles, approximately thirty percent of individuals were categorized in the discordantly high RC/low LDL-C group, and 302 patients were categorized in the discordantly low RC/high LDL-C group. KM curves showed a significant difference in the incidence of 3-year TVF among individuals with concordant/discordant RC levels (*p* = 0.025), and the individuals with discordantly low RC/high LDL-C had the lowest incidence of TVF (Fig. 5). To assess the reliability of these findings, discordance analysis was repeated using the definition based on the clinical cutoff value. The study population was divided into four groups: Concordant high RC/LDL-C group (RC ≥0.8 mmol/L, LDL-C ≥2.6/1.8 mmol/L); Discordantly high RC/low LDL-C group (RC ≥0.8 mmol/L, LDL-C <2.6/1.8 mmol/L); Discordantly low RC/high LDL-C group (RC <0.8 mmol/L, LDL-C ≥2.6/1.8 mmol/L); Concordant low RC/LDL-C group (RC <0.8 mmol/L, LDL-C <2.6/1.8 mmol/L).

The KM curves showed that individuals with discordantly high RC/low LDL-C levels had the highest incidence of TVF events over the 3-year follow-up (*p* = 0.004; *p* = 0.009). Compared with the concordantly low RC individuals, the HR (95% CI) for those who had discordantly high RC levels was 2.65 (1.52–4.64) for TVF risk (*p* < 0.001). However, there was no significant increased risk in patients with discordantly high LDL-C compared with the concordant low RC/LDL-C group [HR= 1.30 (0.89–1.90), *p* = 0.173]. When different cutoff points were set, similar results were obtained [Concordant low RC/LDL-C group vs. discordantly high RC/low LDL-C group: HR= 2.02 (1.33–3.07), *p* < 0.001; Concordant low RC/LDL-C group vs. discordantly low RC/high LDL-C group: HR= 1.20 (0.70–2.08), *p* = 0.508].

### 3.5 Prediction Values of RC

**Fig. 6.**
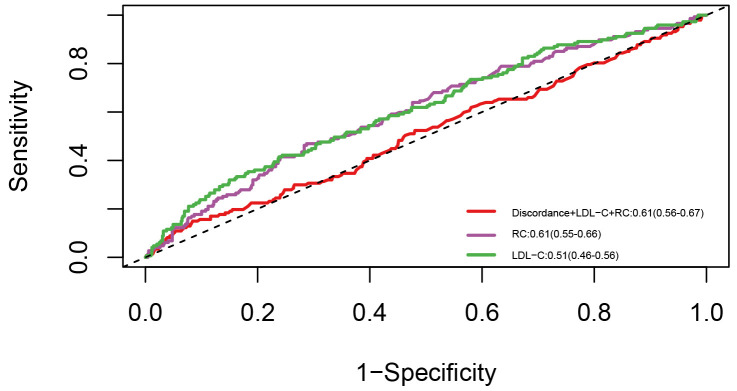
**ROC curve**. Abbreviations: RC, remnant cholesterol; LDL-C, low-density lipoprotein cholesterol; ROC, receiver operating characteristic.

To further assess the predictive value of RC, ROC analysis was conducted based on the Cox regression model (Fig. 6). For reference, when LDL-C level was applied to the cohort of patients diagnosed with ISR, the AUC value (95% CI) was 0.51 (0.46–0.56). Further, RC level significantly outperformed LDL-C level in predicting the risk of TVF [0.61 (0.55–0.66) vs. 0.51 (0.46–0.56), *p* = 0.014]. In this ROC analysis, the discordance of RC/LDL-C, calculated as the percentile distance between RC and LDL-C, was considered as a continuous variable. When RC, LDL-C, and the discordance of RC/LDL-C were incorporated into the model, the predictive ability of the model for TVF was further enhanced [0.61 (0.56–0.67) vs. 0.51 (0.55–0.66), *p* = 0.007; IDI: 0.11, 95% CI: 0.05–0.18; NRI: 0.09, 95% CI: 0.05–0.16].

## 4. Discussion

This retrospective study explored the association between RC levels and TVF among patients diagnosed with ISR who underwent re-PCI. The main findings were as follows: (1) RC levels were associated with TVF risk, (2) the risk of TVF related to RC was more pronounced in patients with LDL-C levels <1.8 mmol/L, (3) discordantly high RC was associated with an increased risk of TVF, and (4) RC level combined with RC/LDL-C discordance improved the prediction of TVF risk in patients with ISR.

Comprising the triglyceride-rich lipoproteins (TRL), RC is composed of the cholesterol content in intermediate-density lipoprotein cholesterol and very-low-density lipoprotein cholesterol under fasting conditions and the cholesterol content in chylomicrons under non-fasting conditions [28, 29]. Similar to LDL-C, these particles can enter the arterial endothelial layers and participate in the initiation and advancement of atherosclerosis [30]. These remaining particles contain around 40 times more cholesterol per particle compared to LDL-C, which might imply a greater atherogenic potential [31, 32, 33]. In contrast to LDL-C, these remnants do not necessitate oxidation before uptake by macrophages, which could cause foam cell formation and accelerate inflammation [34, 35]. Caused by an amplified reendothelialization response to the intimal impairment after stent implantation, inflammation is a key driver of ISR [36]. Future research is needed to clarify the mechanism by which the TRL activates the excessive hyperplasia response in the local lesion within the stent segment, thereby creating a microenvironment conducive to restenosis. Furthermore, the continuous integration of TRL into the arterial wall following DES implantation not only facilitates the transition from in-stent neointima to neo-atherosclerosis but also injures the stability of plaques, thereby lowering the treatment effect of re-PCI and influencing long-term prognosis [37]. Considering these mechanisms that make triglyceride-rich lipoproteins a potential risk factor, previous research has focused on the association between RC level and cardiovascular clinical outcomes. Cohort studies have shown that remnant lipoprotein levels could be a predictive factor for the risk of ASCVD [38]. For individuals with acute coronary syndrome, an elevated RC level was significantly associated with increased risks of ischemic events, cardiac, and all-cause mortality [39, 40, 41]. In a pooled analysis, Palmerini *et al*. [42] revealed that 25% of patients with ISR experienced MI. Furthermore, patients with ISR had an increased risk of recurrent ISR and revascularization [43, 44]. The findings of this study suggest that elevated RC levels may be a potential target related to the cardiac risk in individuals with ISR beyond traditional risk factors.

Characterized by neointimal tissue proliferation and plaque progression, the incidence and prognosis of ISR are associated with individual characteristics, including disease history and clinical symptoms [45, 46]. For example, diabetic individuals had an almost 9% rate of ISR, which was higher than that of individuals without diabetes [47]. Therefore, to verify the robustness of using RC as a prediction factor, its predictive value for clinical outcomes in subgroups was assessed. The results of this study showed that RC-related risk persisted among different subgroups (age, sex, diagnosis, hypertension, and diabetes). Previously, Yang *et al*. [48] discovered that prolonged exposure to elevated RC levels is also related to an increased likelihood of developing other diseases equivalent to ASCVD, such as diabetes and hypertension [49]. This interaction provides a new perspective for evaluating the role of RC in primary and secondary prevention. In the early stage of some chronic diseases, the concentration of TRL might be a potential triggering mechanism. In addition to the clinical disease background, ISR was also classified into different types based on plaque characteristics [37]. Considering the atherosclerotic potential of RC, the RC level may deserve earlier attention in individuals with vulnerable plaques but no ischemia symptoms. For those with relatively stable lesions, early interruption of exposure to high TRL may delay the need for revascularization [3]. For patients with ISR who have already suffered from compromised blood flow, a more stringent RC threshold standard based on lesion characteristics may further improve their prognosis.

Interestingly, the results of subgroup analysis showed that individuals with satisfactory LDL-C control (<1.8 mmol/L) had a greater RC-related risk of TVF than individuals with greater LDL-C values. This tendency differed in previous research, which focused on different study populations and clinical cutoff values [50, 51, 52]. According to consensus and guidelines, LDL-C remains the primary lipid-lowering target and improves clinical outcomes after PCI [53, 54, 55]. In this study, most patients underwent statin therapy after PCI, and the difference in RC-related risk in individuals with high or low LDL-C levels may be explained by that the association between TVF residual lipid risk was partly weakened or overlapped among patients who experienced greater LDL-C lowering. Considering that most patients experienced a change in adverse atherogenic lipid profiles after post-PCI meditation [56], the RC-related risk under different LDL-C lowering levels after PCI needs to be further researched to clarify this hypothesis.

Considering the residual risk of LDL-C-lowering therapy, previous research has focused on the discordance between LDL-C and other atherogenic lipid profiles and their influence on clinical outcomes [57, 58]. As a potential lipid-lowering target, the current cutoff value of high RC has varied among studies and lacks a standard definition [59]. Utilizing several different definitions, the discordance analysis showed that individuals with concordantly high RC/low LDL-C, but not low RC/high LDL-C, had a higher follow-up TVF risk. Consistent with a previous study, this finding reaffirmed the predictive value of RC [57]. It is worth noting that a high RC level rather than a high LDL-C level appeared to be associated with an increased TVF risk. Several factors may have contributed to this result. Researchers have suggested that the biological mechanism of increased RC levels may involve increased secretion and lipolysis dysfunction of triglyceride-rich lipoproteins [60]. Increased RC is related to a higher frequency of insulin resistance and pro-inflammatory status, which further amplifies its effect on cardiovascular events [61, 62]. As a type of stent failure event, most patients may undergo routine LDL-C-lowering therapy after the first PCI [63]. However, statins, ezetimibe, and PCSK9 inhibitors do not significantly lower RC levels [49]. Thus, when LDL-C is adequately controlled, patients with high RC levels may have a residual lipid risk and poor prognosis.

Based on the findings of this study, we conclude that RC may be a risk-enhancing factor for patients with ISR. The predictive model for TVF events, which includes baseline RC level and its post-PCI trajectory, may improve cardiac risk assessment. Furthermore, although the effect of RC-lowering therapy requires validation, patients with an optimal LDL-C level are more likely to benefit from these therapies.

## 5. Strengths and Limitations

This study has several limitations. First, owing to its retrospective characteristic, causality could not be clearly established. Second, the direct measurement of RC is difficult to conduct in standard clinical practice; therefore, this study utilized the calculated RC method, which measured the cholesterol content of all lipoproteins that were not HDL or LDL. Thus, there may be a discordant residual risk between the calculated RC and measured RC regarding the effect of triglyceride levels. Third, during the extended follow-up period, the fluctuation of the RC level was not tracked. Considering that statin therapy could lower RC to some extent, the degree of reduction or increase in RC level should be considered in future analyses.

## 6. Conclusion

For patients diagnosed with ISR, RC levels were associated with increased TVF risk after PCI. RC levels emerged as an independent risk factor for TVF among individuals with ISR. This relation remained among individuals with optimal LDL-C levels (<1.8 mmol/L). These findings reclaimed the clinical value of RC and its potential as a lipid-lowering therapeutic target. The discordant analysis and incremental predictive value of RC also show the limitations of the present cardiac risk management with a single lipidic indicator. Therefore, further research was needed to explore optimized management of a comprehensive lipid profile to improve the prognosis.

## Availability of Data and Materials

The datasets generated or analyzed during the current study are available from the corresponding author, subject to a reasonable request.

## Supplementary Material

Supplementary material associated with this article can be found, in the online version, at https://doi.org/10.31083/RCM43867.
