# Supplementary material for: Remnant Cholesterol as a Predictor of Target-Vessel Failure in Patients With In-Stent Restenosis
Source: Rev Cardiovasc Med. 2025 Nov 26;26(11):43867. doi: 10.31083/RCM43867 (PMC12680994; doi:10.31083/RCM43867)
Supplement: Supplementary file 1 [file 2153-8174-26-11-43867-s1.docx]

**Supplementary Table1.** Baseline characteristics

| **Variables** | **Total**  **(n = 836)** | **Q1**  **(n = 209)** | **Q2**  **(n = 209)** | **Q3**  **(n = 209)** | **Q4**  **(n = 209)** | ***P*** |
| --- | --- | --- | --- | --- | --- | --- |
|  |  |  |  |  |  |  |
| NT-pro BNP>300pg/ml, (%) | 423 (50.6) | 81 (38.8) | 110 (52.6) | 140 (67.0) | 92 (44.0) | **<.001** |

**Supplementary Table 2.** Associations between RC level and TVF in Cox regression model regression model

| **Variables** | **Univariate Cox**  **regression model** | |  | **Multivariable Cox**  **regression model** | |
| --- | --- | --- | --- | --- | --- |
|  | **P** | **HR (95%CI)** |  | **P** | **HR (95%CI)** |
| **Continuous** | | |  |  | |
| RC level (mmol/L), per 1 SD increase | <0.001 | 1.19 (1.09 ~ 1.29) |  | 0.024 | 1.44 (1.05 ~ 1.97) |
| **Categorical** |  |  |  |  |  |
| Q1 quartile | (Reference) | 1.00 |  | (Reference) | 1.00 |
| Q2 quartile | 0.096 | 1.58 (0.92 ~ 2.70) |  | 0.123 | 1.50 (0..91 ~ 2.62) |
| Q3 quartile | 0.042 | 1.73 (1.02 ~ 2.93) |  | 0.125 | 1.53 (0.89 ~ 2.63) |
| Q4 quartile | <.001 | 2.74 (1.67 ~ 4.49) |  | 0.003 | 2.37 (1.32 ~ 4.27) |

Adjusted for sex, age, diabetes, hypertension, Lp(a), RBC, triglycerides, and Scr. RBC, red blood cell; RC, remnant cholesterol; Scr, Serum creatinine;
